# Influence of Marital and Parental Status on Public Reactions to Stuttering in Chile: A Socio-Demographic Study

**DOI:** 10.3390/ijerph22111662

**Published:** 2025-11-02

**Authors:** Yasna Sandoval, Carlos Rojas, Bárbara Farías, Gabriel Lagos, Ángel Roco-Videla, Arnaldo Carocca, Goncalo Leal

**Affiliations:** 1 Department of Health Rehabilitation Sciences, University of Bío-Bío, Chillán 3780000, Chile; ysandoval@ubiobio.cl; 2 Dirección de Desarrollo y Postgrados, Universidad Autónoma de Chile, Santiago 8920112, Chile; anroco@unap.cl; 3 Vicerrectoría de Investigación e Innovación, Universidad Arturo Prat, Iquique 1110939, Chile; 4 Department of Speech Therapy, University of Concepción, Concepción 4070386, Chile; acarocca@udec.cl; 5 Speech Care Center, 1600-100 Lisboan, Portugal; goncalo.tf@gmail.com

**Keywords:** stuttering, public reactions, opinion, communication disorder, married, parents

## Abstract

Stuttering is a communication disorder that significantly impacts individuals’ quality of life. This study examines public reactions towards stuttering within the Latin American context, specifically in Chile, using the Public Opinion Survey of Human Attributes-Stuttering. Data were collected from 400 adults, revealing that married individuals and parents exhibit heightened sensitivity and concern towards stuttering, especially regarding close family members. For instance, 56.86% of married respondents expressed worry about a stuttering sibling, contrasting sharply with only 27.18% of single respondents. Moreover, parents were notably anxious about stuttering in their family. This study underscores the significant role of marital status and parental responsibilities in shaping public attitudes towards stuttering. Additionally, it emphasizes the influence of family-centric values, advocating for the need for comprehensive educational initiatives to combat prevailing stigma towards individuals with stuttering.

## 1. Introduction

Stuttering is a communication disorder characterized by involuntary disruptions in the fluency of speech, affecting approximately 5% of children aged 4 years old, with varying rates of persistence into adulthood. While several studies indicate that stuttering can exhibit a natural course of recovery in some individuals, it remains a concern over time for many, transitioning into adulthood where persistence of the disorder is often observed [1,2]. It is characterized by repetitions, prolongations, broken words, blocks, circumlocutions, and excessive physical tension. It may be accompanied by cognitive, social, and emotional components, which are predominantly negative and can impact the individual’s quality of life. Recent research has shown that public awareness and knowledge about stuttering can influence the attitudes presented towards individuals with this diagnosis [3]. In general, negative attitudes toward stuttering—such as perceiving individuals as nervous, and insecure—have prompted efforts to increase public awareness and education on the topic, thereby reducing the associated social stigma [4]. However, there is still limited information available on this subject. In this regard, some studies indicate that awareness campaigns and public knowledge about stuttering promote a better social environment by providing greater understanding and eliciting positive reactions toward individuals with this diagnosis [4,5,6,7,8,9].

Public reactions to stuttering encompass a complex interplay of social constructs, psychological factors, and demographic variables. For example, individual with higher levels of education tend to have more positive attitudes toward stuttering, often due to increased exposure to health-related topics, which fosters greater understanding and diminishes stigma. In addition, married individuals, especially parents, may show heightened empathy toward family members who stutter [10,11]. Research indicates that parental concern for children who stutter reflects deeper emotional connections and influences perceptions and attitudes toward stuttering [4,6]. On the other hand, while some studies show that men often face greater stigma due to societal pressures regarding communication efficacy, other findings suggest that socio-demographics contexts can equalize perceptions of anxiety related to stuttering across genders [4,6]. This theoretical framework seeks to elucidate how these elements influence public perceptions and attitudes toward stuttering, impacting the experiences of people with stuttering. In this analysis, it is imperative to highlight the diverse sociodemographic variables that shape these reactions, such as gender, age, race, education, and cultural background, which cumulatively contribute to the social aspects around stuttering. This complexity is underscored by findings indicating that this factors values play a crucial role in shaping public attitudes, as noted by Amura et al. [11], who identified how Chilean idiosyncrasy values intersect with socio-structural determinants of health, resulting in barriers that can exacerbate existing inequities among Hispanic/Chilean patients [11,12,13].

### 1.1. Socio-Demographic Influences on Reactions to Stuttering

Understanding the socio-demographic context is essential to analyzing public reactions to stuttering. Recent research reveals marked demographic differences in stuttering perceptions, particularly with respect to gender. Studies indicate that men experience higher social anxiety related to stuttering severity, often resulting in increased stigmatization and isolation [12,14,15,16]. Moreover, childhood experiences with stuttering influence long-term self-concept and social attitudes; school-aged children with stuttering often display increased introversion and social withdrawal, driven by negative peer interactions [10]. Additionally, parental attitudes play a crucial role, as a lack of understanding among parents can exacerbate frustration and self-blame in affected children, further reinforcing negative societal perceptions [17]. Collectively, these findings underscore the need to consider socio-demographic factors in efforts to reduce stigma and enhance support for individuals with stuttering.

Socio-demographics and cultural factors have an important influence on how the public perceives stuttering. Research has shown that attitudes toward stuttering can vary significantly across different cultures, particularly in preschool children [9,18]. In cultures that value communal and social interactions, stigma related to stuttering may be more noticeable, resulting in increased anxiety for both children and adults who stutter. In Latino and Chilean communities, cultural values such as familism and respect are particularly influential in shaping attitudes toward stuttering and affecting relationships [19]. These attributes can sometimes alleviate stigma but may also intensify it, depending on the context [13,20]. Familism highlights the value of family support, while respect emphasizes the importance of treating others well. Together, these values can create a more accepting environment [10,19]. Overall, these factors of heritage significantly impact how individuals with stuttering understand their condition and how they are perceived by society, influencing whether they receive support or face marginalization [21].

In exploring how Chilean idiosyncrasy influence reactions to stuttering, it is essential to consider the region’s diverse social constructs, norms, and values, which shape public perceptions and attitudes towards individuals with stuttering. Socio-demographic characteristics in Latin America (and in Chile)—ranging from familial collectivism to views on disability—play significant roles in how stuttering is perceived and reacted to in various social contexts. One key aspect of Chilean culture is the emphasis on familial and community relationships. Family is often viewed as a central support system, and as a result, responses to stuttering may be heavily influenced by familial attitudes. Parents and family members who understand stuttering may foster a more positive social environment, which can mitigate stigma. Conversely, if family attitudes reflect societal stigmas, children with stuttering might experience increased pressure and isolation. This phenomenon aligns with findings indicating that parental involvement in managing a child’s stuttering significantly influences the child’s self-esteem and coping mechanisms [10,17]. Research demonstrates that temperament is a significant factor in the impact of stuttering, as it is closely related to a child’s experiences. The environment in which a child is raised plays a crucial role in shaping their temperament and, subsequently, their experiences with stuttering. Studies have shown that children who stutter exhibit varying degrees of emotional reactivity and regulation, which can influence their social interactions and overall mental well-being [22,23,24,25]. Understanding the interplay between temperament and the social context is essential for developing supportive interventions that address the unique needs of children with stuttering.

Geographic differences significantly influence public attitudes toward stuttering, indicating that even within culturally similar contexts, perceptions can vary greatly due to geographic and socio-economic factors [26]. These variations may arise from differing levels of awareness, healthcare access, and educational resources related to speech disorders across regions [21]. For instance, areas with robust educational initiatives and awareness campaigns may foster a more accurate understanding of stuttering, thereby promoting more positive societal attitudes and reducing stigma. In contrast, regions with limited access to information and resources may perpetuate misconceptions about stuttering, leading to increased stigmatization [6,26,27,28]. Tailoring awareness campaigns to educate the public about the complexities of stuttering is essential for altering these perceptions and creating a more inclusive society [28]. This is particularly relevant within Latino (Chilean) cultures, which often emphasize strong familial support systems and community cohesion. These attributes can play a dual role, either alleviating or intensifying stigma surrounding stuttering, contingent upon the attitudes held by family members and peers within these networks [27]. For example, when family attitudes are supportive and informed, they can create a nurturing environment that fosters acceptance. Conversely, if stigma is prevalent within families, it can exacerbate feelings of isolation and anxiety for individuals who stutter. By addressing these geographics factors within public education strategies, communities can work towards more compassionate and understanding environments for individuals with stuttering.

Furthermore, the degree of public awareness and education about stuttering significantly varies throughout Latin America, affecting attitudes towards people with stuttering [19,29,30]. In regions where educational resources are scarce, misunderstandings about stuttering prevail, often leading to stigmatization. Studies have shown that a lack of knowledge about the nature of stuttering among the general public reinforces negative perceptions [9,10]. Hence, educational initiatives aimed at enhancing public understanding of stuttering are crucial for altering societal attitudes and reducing stigma.

Social norms and cultural taboos regarding disabilities may also affect public responses to stuttering. In some contexts, there is a prevalent belief that disabilities or speech disorders like stuttering are personal failures, thereby reinforcing stigma [31,32]. A growing body of socio-cultural insight suggests that these beliefs may affect how individuals with stuttering are treated and, consequently, their self-identity and social integration. Furthermore, structural factors, such as economic conditions and access to healthcare resources, complicate this dynamic by influencing the availability of speech therapy and support services for individuals who stutter [4].

Research has highlighted notable regional differences in attitudes toward stuttering within Latin America, indicating that cultural perceptions influenced by historical, social, and economic contexts significantly shape how stuttering is viewed and addressed across various countries [19,30]. For example, studies have shown that these cultural nuances can lead to substantial variations in public perception and treatment of individuals who stutter [33]. In Chile, where family-centric values and social networks play a crucial role, the attitudes towards stuttering can be markedly different from those in neighboring countries, reflecting unique socio-cultural dynamics [34,35]. These findings underscore the necessity of conducting context-specific analyses to thoroughly understand public attitudes and to effectively tailor interventions aimed at reducing stigma associated with stuttering [4,6,36]. By acknowledging these regional differences, stakeholders can develop more effective educational programs and support systems that resonate with the local population’s values and beliefs.

Socio-cultural beliefs about identity and communication also contribute to how stuttering is viewed in Chile. Effective communication is often highly valued, and disruptions in fluency, such as stuttering, can trigger negative judgments about a person’s competence or social value. In societies where collectivism is emphasized, individuals with stuttering may face increased criticism or stigma, diminishing their self-worth and promoting feelings of anxiety related to speaking in public or social situations [37]. Additionally, this pressure may lead to individuals internalizing negative societal views, hindering their ability to seek help or convey their experiences effectively.

### 1.2. Psychological Implications and Assessment of Public Attitudes

The psychological implications of societal attitudes towards stuttering cannot be overlooked. Stuttering can often lead to psychological burdens such as anxiety and depression, particularly in environments where there is a lack of awareness and acceptance [38]. Evidence indicates that a significant percentage of adults with stuttering may develop comorbidities, particularly in the realm of mental health, with social anxiety being one of the most prevalent concerns. Research suggests that between 22% and 60% of adults with stuttering meet the criteria for social anxiety disorder [39]. These elevated rates of anxiety can adversely affect therapeutic outcomes, as many adults may avoid seeking treatment due to their extreme anxiety or social phobia [14,15]. Furthermore, studies have demonstrated a correlation between the severity of stuttering and an increased risk of emotional deterioration, which subsequently impacts the effectiveness of therapeutic interventions. This highlights the necessity for an integrated approach that addresses not only the speech fluency but also the emotional and psychological factors associated with stuttering, in order to achieve improved treatment outcomes [22,40].

A pivotal tool within this framework is the Public Opinion Survey of Human Attributes for Stuttering (POSHA-S), originally developed by St. Louis et al. [41] to measure public attitude toward stuttering and provide valuable insights into societal beliefs regarding this speech disorder. Over time, this instrument has been successfully applied across diverse contexts; it has been translated into various languages and culturally adapted [3], yielding important comparative data that illuminates how different cultural backgrounds shape attitudes toward stuttering [7,25,42]. Not only does POSHA-S facilitate the evaluation of attitudes towards stuttering, but also it allows comparisons with other human attributes that may invoke stigma or acceptance in various contexts. The instrument comprises distinct sections that delve into how survey participants perceive stuttering and their anticipated reactions when encountering individuals with stuttering. The validity and reliability of POSHA-S further equip it to be utilized effectively in different socio-demographics contexts [43,44], particularly in examining the idiosyncrasies within Latin American communities. Recent studies have demonstrated the effectiveness of using this tool to bridge gaps in understanding between different populations regarding their attitudes towards stuttering [45].

### 1.3. The Present Study

Latin American idiosyncrasies may significantly influence public reactions to stuttering—through family attitudes, societal values relating to communication, misconceptions about disabilities, and regional differences in perception. Therefore, this study specifically examines public reactions to stuttering as measured by the Chilean version (translated and culturally adapted by Sandoval et al. [3]) of the Public Opinion Survey of Human Attributes for Stuttering. The primary objective is to analyze how socio-demographics factors how marital and parental status, shape these public responses in Chile. Rather than investigating the underlying beliefs that might drive these responses, the study prioritizes the observation of direct, immediate attitudes—how individuals react in social settings toward people with stuttering. Given the complex socio-demographic landscape, which includes variables such as marital and parental status, this approach offers a practical lens through which to examine the interactions between these factors. Ultimately, this study aims to contribute to a broader understanding of how negative public perceptions can adversely affect the quality of life of individuals with stuttering. Addressing these socio-demographic factors through tailored educational campaigns and community engagement is essential for fostering more inclusive environments.

## 2. Materials and Methods

### 2.1. Participants

The target population consists of Chilean adults from the Bío-Bío region, recognized as a representative socio-demographic and economic area according to the Chilean national statistics index. For calculating the sample size, recommendations for psychometric property investigations indicate an estimation of 5 to 10 participants per instrument item, leading to a necessary sample size of 385 participants, which was increased to 400 to enhance study power [46]. The sample was a non-probabilistic incidental type, comprising 400 participants (150 men and 250 women) recruited by the principal investigator from the Bío-Bío region of Chile. Participants ranged in age from 18 to 70 years, with the majority being under 30 years (51.25%). The sample was predominantly female (62.50%) and married (74.50%), with many participants not being parents (63%). In addition, more than half of the participants had over 12 years of education (51.25%), and most identified as Catholic (40.25%). The average age was 35.09 (SD: 15.11) years for men and 32.43 (SD: 14.43) years for women, while the mean level of education was 13.64 (SD: 3.26) years for men compared to 14.00 (SD: 3.54) years for women.

### 2.2. Eligibility Criteria

Participants in the survey were required to meet the following conditions. Inclusion criteria: (1) individuals of both sexes (male and female); (2) individuals aged 18 years or older; (4) Chilean or Latino nationals; (3) individuals who signed the informed consent form. Exclusion criteria: (1) individuals diagnosed with stuttering; (2) professionals with degrees in speech pathology or speech therapy; (3) students of speech pathology or logopedics; (4) individuals with cognitive impairment, sensory alterations, or communication issues impacting survey responses; (5) individuals who did not complete the survey from excluded subjects were archived in a database created by the responsible researcher, ensuring the confidentiality of personal data and sensitive information in accordance with Chilean laws. Finally, respondents were not required to have any baseline knowledge of stuttering, nor were they asked if they knew anything about the condition prior to the survey.

### 2.3. Instrument

The POSHA-S (created by St. Louis et al. [41]) is an instrument designed to measure public attitudes toward stuttering. However, given the socio-demographics characteristics of the target population, this study employed the version of the POSHA-S that was translated and culturally adapted into Chilean Spanish by Sandoval et al. [3]. POSHA-S (version translated) consists of three sections: (a) demographic section, (b) general section about stuttering related to attributes, and (c) attitudes towards stuttering (beliefs and reactions). Furthermore, it includes instructions for the instrument. The demographic section covers personal details such as gender, age, education, languages spoken, occupation, and marital status. The attributes section presents human characteristics for comparison, allowing for the evaluation of stuttering within the context of other human attributes. The attitudes section combines components of beliefs and reactions. The attributes section is composed of three components: (a) self-identification; (b) amount of knowledge; and (c) impression. The attitudes section consists of components shaped by beliefs and reactions, including beliefs about traits/personality, assistance for individuals who stutter, causes of stuttering, and potential for social and professional success.

The reactions section includes: (1) Social Distance/Sympathy: this dimension gauges the concern individuals express regarding stuttering based on their relationship with the person who stutters. It evaluates comfort levels in interactions depending on social proximity, revealing that greater concern is felt for close relatives compared to more distant acquaintances, reflecting how empathy is influenced by emotional closeness. (2) Accommodation/Help: this dimension assesses the types of assistance individuals believe are appropriate for those who stutter, such as whether they would complete sentences or suggest that the person should “speak more slowly.” It differentiates between supportive behaviors and potentially patronizing actions, capturing societal attitudes towards interactions with people who stutter. (3) Knowledge Source: this dimension explores where individuals acquire their understanding of stuttering, such as personal experiences, media, education, or healthcare providers. This assessment helps identify gaps in knowledge that may contribute to misconceptions and stigma regarding stuttering. To access the Chilean version of the survey and table of reactions, see Supplementary Material S1.

### 2.4. Ethical Considerations

Prior to participating in the survey, eligible individuals were provided with an informed consent form that had been approved by the Ethics Committee of the sponsoring university. Participants carefully reviewed the consent document, addressed any questions they had, and acknowledged that their participation was both voluntary and confidential.

### 2.5. Procedure

The survey was conducted in public spaces, targeting a non-probabilistic sample of 400 participants. Each survey was administered in a face-to-face manner, ensuring direct interaction with participants. Two trained speech-language pathology professionals provided the initial instructions about the survey process to each participant. Importantly, individuals responded independently to the survey questions, maintaining the integrity of their answers. Each participant received an informed consent form to guarantee voluntary participation and to uphold confidentiality throughout the data collection process. The survey took approximately 10 min to complete, with questions reiterated as necessary without prompting responses.

Eligibility criteria were rigorously applied using the demographic section of the survey to ensure that respondents met the necessary qualifications for inclusion in the study. Consequently, 51 individuals were excluded from the final analysis for failing to meet these eligibility criteria. Additionally, 11 participants opted to withdraw from the survey, citing time constraints as their reason for discontinuation. In both cases, these individuals were acknowledged and thanked for their willingness to participate in the study. Thus, the final sample comprised 400 respondents who completed the survey in its entirety and met all specified criteria.

At the conclusion of the survey, an informational pamphlet regarding stuttering awareness was distributed to all participants (Supplementary Material S2). The survey was conducted over a five-week period between January and February 2025. Subsequently, each survey was coded to safeguard the confidentiality of the participants and their responses. Each item was then transferred to a Microsoft Excel spreadsheet for subsequent statistical analysis.

### 2.6. Data Analysis

Statistically significant differences were investigated according to grouping by sociodemographic variables, including biological sex, marital status, level of education, religious belief, age group, and parental status. The Mann–Whitney U test and the Kruskal–Wallis test were employed to determine these differences, with a 95% confidence level and a significance threshold set at a *p*-value of less than 0.05. The results are presented for each question as relative percentage frequencies. Data analysis was conducted using STATA software version 15 (in Spanish).

## 3. Results

The following analysis presents the results obtained from the study on public reactions to stuttering (including social distance/sympathy, accommodation/help, and knowledge source dimensions), detailing the perceptions of the public regarding this speech disorder in Chile. Among the variables analyzed, marital status and parental status (having or not having children) exhibited the greatest number of statistically significant differences in responses, with 6 out of 17 significant differences in each case. In contrast, the other study variables—biological sex, level of education, religious belief, and age group—did not show statistically significant differences across the questions.

Table 1 displays the overall distribution of participants’ responses regarding their perceptions of stuttering in different contexts. With respect to social distance/sympathy, concern is notably higher when a person who stutters is a close relative, especially a sibling (R3, 34.75%) or the participant themself (R4, 59.50%). In contrast, concern is markedly lower when the speaker with stuttering is described as a physician (14%) or a neighbor (6.5%), suggesting that emotional connection plays a significant role in shaping individual perceptions. Impatience (R8, 26.5%) and pity (R10, 13%) appear with moderate to low frequency, indicating an overall perception that is predominantly neutral or empathetic.

Regarding accommodation/help, the majority of respondents (87.25%) indicated that they would treat a person who stutters as if they were speaking normally, reflecting a tendency towards humane and respectful interaction with individuals affected by this condition. However, 31.5% (R7) admitted to having completed the words of a person who stutters, and 44% (R11) reported having suggested that the speaker should relax. These behaviors may be interpreted as paternalistic interventions or as manifestations of the respondents’ own anxiety when interacting with a person with this condition.

Lastly, in relation to knowledge source, the participant’s personal experience (R12, 51.25%) and the internet (R15, 56.5%) emerged as the main sources of information. This finding aligns with a global trend in which individuals highly value online information, especially given the large volume available on the subject [47]. Nevertheless, while expert testimony continues to be crucial in matters of health—a trend that appears to be gradually declining in practice—formal education (R16, 43%) and traditional media (R13–R14) are considered less relevant as sources of information on stuttering.

Table 2 compares the responses of participants according to marital status. Regarding social distance/sympathy (R1–R4), married respondents exhibit greater concern in all contexts (medical, neighbor, sibling, and self), especially toward their sibling (R3, 56.86% vs. 27.18%) and themselves (R4, 69.61% vs. 56.04%). This pattern of heightened concern underscores the impact of familial relationships on the perception of stuttering, indicating that emotional closeness fosters increased empathy. In terms of accommodation/help, married respondents are more likely to suggest interventions [19], such as “relax,” than single respondents (R11, 54.9% vs. 40.27%). This finding may reflect a more controlling or protective approach assumed by married individuals, who attempt to assist the person who stutters in a manner they perceive as helpful, albeit not necessarily appropriate. Finally, regarding knowledge source, single respondents report a greater reliance on the internet (R15, 64.43% vs. 33.33%).

Table 3 details the differences in participant responses according to parental status. Concerning social distance/sympathy, parents exhibited greater concern about stuttering among their close ones, especially regarding a sibling (48.65% vs. 26.59%). This finding emphasizes the heightened emotional sensitivity that parents may have toward stuttering, which could be related to deeper familial bonds. Additionally, regarding accommodation/help, parents displayed a higher level of unease or restlessness on the subject (R8, 35.14% vs. 21.43%). This may suggest that when confronted with situations involving stuttering, parents experience a greater emotional burden than non-parents. In terms of intervention (R11), the results indicate that parents are more likely to suggest that the person who stutters “relax” (57.43% vs. 36.11%). Finally, with respect to the knowledge source (R15), the use of the internet is considerably more prevalent among non-parents (67.06% vs. 35.51%).

The analyses presented in the tables reveal that both marital status and parental status significantly influence individuals’ perceptions and responses to stuttering. Concern and sensitivity are more pronounced among married individuals and parents, reflecting a more empathetic approach toward family members and close contacts who experience this speech disorder. The predominant reliance on personal experience and the internet as sources of knowledge about stuttering underscores the need for educational initiatives aimed at informing and reshaping public attitudes on the matter. Therefore, it is essential to develop campaigns that transform existing negative perceptions and foster a more understanding and inclusive environment for people who stutter.

## 4. Discussion

### 4.1. Socio-Demographics Influences on Public Stuttering Perceptions

The study titled “Influence of Marital and Parental Status on Public Reactions to Stuttering in Chile: A Socio-Demographic Study” provides critical insights into how socio-demographic characteristics influence public perceptions of stuttering within the Chilean context. This work elucidates the nuanced reactions individuals display toward those who stutter, particularly highlighting the roles of marital status (married versus single) and parental status (having children versus not having children). These demographic variables, intertwined with local cultural traits, profoundly impact behaviors and perceptions in various health-related contexts [48]. For instance, married individuals and parents may exhibit greater empathy and concern for stuttering due to the inherent responsibilities and emotional bonds associated with family relationships. This suggests that family dynamics and societal expectations surrounding marital and parental statuses can significantly shape how stuttering is perceived and responded to by the public. Our findings reinforce the notion that socio-demographic characteristics are pivotal in understanding varying attitudes toward stuttering, ultimately calling for culturally sensitive interventions that address these complexities in public perceptions.

Family relationships, whether through marriage or parenthood, significantly influence health perceptions and attitudes toward various conditions, including stuttering. Interpersonal bonds enhance responsibility for both personal well-being and the welfare of loved ones, promoting greater awareness of health matters and healthier behaviors [49]. Moreover, the roles of spouses and parents often cultivate empathy toward communication challenges, as indicated by increased sensitivity when stuttering is exhibited by family members [14,15].

From a socio-demographic perspective, family networks play a crucial role as bonding resources that offer emotional support and facilitate the sharing of health-related information within communities. According to Putland et al. [50], these networks function at various levels—bonding, bridging, and linking—each contributing to enhanced health outcomes for individuals within those communities. This interplay highlights how a supportive family environment can diminish social stigma associated with health issues, including stuttering, by fostering positive and nurturing contexts. Such dynamics are consistent with ecological theories that emphasize the necessity of cultivating supportive social environments to counteract negative health perceptions and behaviors [51]. By examining the socio-demographic characteristics that underlie these family dynamics, we can gain insights into how social capital influences attitudes and responses toward health conditions.

### 4.2. The Role of Family and Idiosyncrasy in Attitude Formation

Moreover, the intersection of family roles with cultural expectations plays a significant role in modifying attitudes. In Latin American contexts, where strong familial values and closeness are prevalent, marital and parental experiences influence individuals’ perceptions of stuttering. The socio-demographic concept highlights the importance of emotional and communicative support from long-term familial relationships in shaping attitudes toward stuttering. Such support enhances empathy for individuals who stutter and helps mitigate the negative social pressures associated with the condition. Research indicates that socio-demographic factors, including marital and parental status, significantly influence these family dynamics. Tailored family-based interventions have been shown to effectively reduce stigma and promote health equity among those affected [52]. By recognizing the role of socio-demographic factors, we can better understand how to create supportive environments that foster acceptance of stuttering.

Furthermore, evidence suggests that effective communication within families, bolstered by mutual support, is critical for shaping adaptive health behaviors and coping strategies. When family members collaborate to provide understanding and accurate information, they cultivate an environment that is more accepting and less judgmental of speech disorders. This comprehensive, supportive approach is essential in interventions aimed at mitigating the adverse impacts of stuttering, ultimately improving the quality of life for affected individuals [11,36].

One of the most significant findings of this study is that Chilean adults tend to react differently depending on their relationship with the person who stutters (social distance/sympathy dimension). The results indicate that greater concern is expressed when stuttering is exhibited by close members of the respondent’s social circle—such as siblings or even the respondents themselves—rather than by more distant figures, such as doctors or neighbors. This finding is consistent with existing literature suggesting that familial relationships (very important aspects for Latin American culture) profoundly influence the perception of speech disorders [19,29,30].

Familiarity with people who stutter who are not family members, such as a sibling, has been associated with more positive and empathetic attitudes [53,54], so the differences observed in the research may not be due solely to the content of the question (the sibling), but to the fact that people with children or in marital relationships may have developed greater sensitivity to interpersonal care given their status. Although the POSHA-S question refers to a sibling, the interpretation of the differences between married/with children and single/without children should not be limited to the figure of the sibling but considered in the broader context of relational experience and empathic development [54,55].

Empathy is influenced by the nature of the relationship, with emotional closeness fostering a deeper understanding of the complexities of stuttering [56]. While this study did not specifically measure empathy, existing research suggests that family dynamics play a critical role in shaping perceptions of stuttering. For example, Rocha et al. [10] found that the perceptions of Portuguese parents regarding stuttering significantly impact their children’s self-view. This highlights how familial responses can help shape self-image in individuals who stutter, emphasizing the importance of family support in navigating the challenges posed by the disorder.

Furthermore, the role of culture is fundamental. In many Latin American communities, family values such as familism and respect significantly influence the understanding and attitudes toward stuttering. These values can act as a double-edged sword: they may mitigate stigma by creating a supportive environment, or they may exacerbate it when familial perceptions of stuttering are negative. When parents and other family members possess a well-informed understanding of stuttering, they can cultivate a more compassionate and positive atmosphere, which in turn enhances the self-esteem and self-efficacy of the person who stutters [2,57]. Conversely, negative familial attitudes that associate stuttering with weakness or incompetence can reinforce social marginalization and worsen the individual’s condition. This observation aligns with previous studies indicating that family attitudes—especially those of Latinas—are key in determining an individual’s capacity to cope with their disorder [56,58].

### 4.3. Gaps in Professional Knowledge and the Need for Educational Interventions

Regarding the “accommodation/help” dimension evaluated in the survey, factors such as age and educational level further complicate responses toward individuals who stutter. Although no statistically significant differences were observed in these items, the survey results suggest that many Chilean professionals—including educators and healthcare workers—lack sufficient information about stuttering. This knowledge gap limits the quality of support they can offer to individuals who stutter. This issue is critical, as a lack of understanding among authority figures can exacerbate isolation and negatively impact the self-esteem of those who stutter, ultimately compromising their emotional well-being [40,59]. Consistent with findings from other studies [10], our research highlights that many Latin American educators, rather than creating a supportive environment, may inadvertently perpetuate misunderstanding and stigmatization. Therefore, targeted training is essential. Programs that integrate instruction on linguistic diversity and fluency disorders should be implemented in the education of future Chilean professionals, ensuring that the next generation of educators and healthcare providers is adequately equipped to deliver effective and compassionate support.

In turn, the lack of knowledge in the Chilean population regarding the nature of stuttering (knowledge source dimension) represents a significant obstacle. Despite the existence of some local awareness campaigns, ignorance and misconceptions persist, potentially perpetuating stigma. Education emerges as a crucial tool for changing these perceptions, as educational initiatives have been shown to transform attitudes toward stuttering and, consequently, improve the social environment for those affected [60]. Although awareness campaigns are in place, persistent negative attitudes indicate that the effectiveness of these initiatives must be critically evaluated. Data suggest that individuals who lack accurate information about stuttering are more likely to contribute to stigmatization [61,62]. Highlighting the connection between education and stigma reduction is not only relevant but essential. In a study by Haitani [63], it was found that well-structured educational programs can change erroneous perceptions of stuttering and, thereby, alter the social interaction dynamics experienced by individuals with this condition.

As future directions, first, it is recommended to conduct longitudinal research that recruits participants from various regions across Latin America to observe long-term changes in perceptions of stuttering following educational interventions. Second, this study underscores the importance of exposure to information about stuttering—whether derived from personal experiences or obtained through online resources—in shaping public attitudes. Such exposure fosters more positive and empathetic perceptions, indicating that informed individuals are likelier to support those who stutter. This finding emphasizes the need for future research to explore effective methods for disseminating information and educating the public, as improving access to knowledge about stuttering could significantly shift societal attitudes and reduce stigma. The tendency of individuals without direct familial ties to seek online information suggests a proactive approach that could be leveraged for more targeted and effective awareness campaigns [57]. Thus, developing culturally tailored campaigns is fundamental; for instance, employing the POSHA-S-CL and other instruments to design awareness initiatives that emphasize local perceptions of stuttering across different communities. Third, it is essential to reinforce an interdisciplinary approach to this issue by integrating psychologists, educators, speech therapists, and community leaders in the creation of programs. Such programs should address stuttering not only from a clinical standpoint but also by focusing on its emotional and social dimensions.

Finally, this research presents several limitations. The adoption of a non-probabilistic sampling technique may limit the applicability of the results to other areas and contexts within Latin America, where attitudes toward stuttering can differ markedly. Furthermore, by concentrating on a particular geographic locale, the study fails to encompass the region’s full cultural diversity. The dependence on self-reported personal experiences and the internet as the primary channels of information may also introduce a bias in self-evaluation, possibly affecting the apparent correlation between beliefs and attitudes toward stuttering. Another critical limitation is noted regarding the statistical analysis, where out of 102 statistical tests conducted across all variables examined, only 12 yielded significant results—accounting for less than 12% of the findings, whereas a 5% significance level would be expected due to random chance alone. This observation suggests that a considerable proportion of the significant findings may have arisen from random variation rather than true effects.

## 5. Conclusions

This study demonstrates that public reactions to stuttering within the Chilean socio-demographic context are significantly influenced by marital status and parental responsibility. Data from 400 adults reveal that married individuals and parents exhibit markedly greater sensitivity and concern when stuttering occurs in close family members. For instance, 56.86% of married respondents expressed concern for a stuttering sibling compared to only 27.18% of single respondents. On the other hand, parents exhibited a higher level of anxiety concerning stuttering among their close family members, with 48.65% expressing concern compared to only 26.59% of non-parents. Additionally, parents were more likely to suggest that individuals who stutter should “relax,” with 57.43% endorsing this advice, in contrast to 36.11% of non-parents. In contrast, other demographic factors—such as biological sex, educational level, religious beliefs, and age group—did not significantly affect these reactions. These findings underscore the pivotal role of family-related values, particularly familism, wherein family cohesion and mutual care shape more empathetic attitudes toward speech disorders. The observed patterns suggest that the deep-seated emotional bonds inherent in marital and parental roles create a framework of responsibility and support that mitigates stigma within the family context. However, the persistence of negative attitudes among the broader public highlights the critical need for comprehensive educational initiatives. Enhancing awareness and providing accurate knowledge about stuttering, particularly through targeted socio-demographic-cultural interventions, may promote more inclusive and respectful perceptions, ultimately improving the quality of life for those who stutter within Chilean communities.

## Figures and Tables

**Table 1:** General distribution of responses POSHA-S-CL (without sociodemographic variables, N:400).

Question	No	Not Sure	Yes
R1 SD/S _I would be concerned if the following person had a stutter … “my doctor.”_	82.25%	3.75%	14.00%
R2 SD/S _I would be concerned if the following person had a stutter … “my neighbor.”_	91.75%	1.75%	6.50%
R3 SD/S _I would be concerned if the following person had a stutter … “my brother/sister.”_	62.50%	2.75%	34.75%
R4 SD/S _I would be concerned if the following person had a stutter … “me.”_	34.75%	5.75%	59.50%
R5 A/H _If I were talking with someone who stutters, I would try to act as if that person were speaking normally._	7.25%	5.50%	87.25%
R6 A/H _If I were talking with someone who stutters, I would make a joke about stuttering._	82.25%	5.00%	12.75%
R7 A/H _If I were talking with someone who stutters, I would complete the person’s words._	61.25%	7.25%	31.50%
R8 SD/S _If I were talking with someone who stutters, I would feel impatient._	65.25%	8.25%	26.50%
R9 SD/S _If I were talking with someone who stutters, I would feel relaxed or comfortable._	26.50%	19.00%	54.50%
R10 SD/S _If I were talking with someone who stutters, I would feel pity for that person._	81.50%	5.50%	13.00%
R11A/H _If I were talking with someone who stutters, I would tell the person to “speak more slowly” or “relax.”_	44.75%	11.25%	44.00%
R12 KS _My knowledge of stuttering comes from… personal experience (me, my family, friends)._	43.00%	5.75%	51.25%
R13 KS _My knowledge of stuttering comes from television, radio, or movies._	53.00%	4.00%	43.00%
R14 KS _My knowledge of stuttering comes from magazines, newspapers, or books._	61.50%	3.75%	34.75%
R15 KS _My knowledge of stuttering comes from the internet._	40.00%	3.50%	56.50%
R16 KS _My knowledge of stuttering comes from my education (school, academia)._	53.75%	3.25%	43.00%
R17 KS _My knowledge of stuttering comes from doctors, specialists, nurses, and others._	69.00%	6.75%	24.25%

**Table 2:** Distribution and Comparison of responses (reaction item POSHA-S-CL) according to Marital Status (Singles 243/Married 102).

Question	Married	No	Not Sure	Yes	*p*-Value
R1 SD/S	Yes	70.59%	1.96%	27.45%	0.0002 ***
	No	86.24%	4.36%	9.40%	
R2 SD/S	Yes	87.25%	0.98%	11.76%	0.0490 *
	No	93.29%	2.01%	4.70%	
R3 SD/S	Yes	41.18%	1.96%	56.86%	0.0001 ***
	No	69.80%	3.02%	27.18%	
R4 SD/S	Yes	24.51%	5.88%	69.61%	0.0119 *
	No	38.26%	5.70%	56.04%	
R5 A/H	Yes	8.82%	4.90%	86.27%	0.6992
	No	6.71%	5.70%	87.58%	
R6 A/H	Yes	83.33%	2.94%	13.73%	0.8140
	No	81.88%	5.70%	12.42%	
R7 A/H	Yes	59.80%	4.90%	35.29%	0.5622
	No	61.74%	8.05%	30.20%	
R8 SD/S	Yes	59.80%	4.90%	35.29%	0.0884
	No	67.11%	9.40%	23.49%	
R9 SD/S	Yes	33.33%	10.78%	55.88%	0.6384
	No	24.16%	21.81%	54.03%	
R10 SD/S	Yes	85.29%	1.96%	12.75%	0.3157
	No	80.20%	6.71%	13.09%	
R11 A/H	Yes	33.33%	11.76%	54.90%	0.0056 **
	No	48.66%	11.07%	40.27%	
R12 KS	Yes	46.08%	5.88%	48.04%	0.4469
	No	41.95%	5.70%	52.35%	
R13 KS	Yes	52.94%	3.92%	43.14%	0.9820
	No	53.02%	4.03%	42.95%	
R14 KS	Yes	61.76%	4.90%	33.33%	0.8645
	No	61.41%	3.36%	35.23%	
R15 KS	Yes	62.75%	3.92%	33.33%	0.0001 ***
	No	32.21%	3.36%	64.43%	
R16 KS	Yes	55.88%	2.94%	41.18%	0.6338
	No	53.02%	3.36%	41.18%	
R17 KS	Yes	63.73%	4.90%	31.37%	0.1213
	No	70.81%	7.38%	21.81%	

* *p* < 0.05 ** *p* < 0.01 *** *p* < 0.001 (Mann–Whitney U test and the Kruskal–Wallis).

**Table 3:** Distribution and Comparison of responses (reaction item POSHA-S-CL) according to Parental Status (Without Children: 252/With Children: 148).

Question	With Children	No	Not Sure	Yes	*p*-Value
R1 SD/S	Yes	75.68%	2.70%	21.62%	0.0051 *
	No	86.11%	4.37%	9.52%	
R2 SD/S	Yes	87.16%	1.35%	11.49%	0.0089 **
	No	94.44%	1.98%	3.57%	
R3 SD/S	Yes	50.68%	0.68%	48.65%	0.0000 ***
	No	69.44%	3.97%	26.59%	
R4 SD/S	Yes	29.73%	4.73%	65.54%	0.0669
	No	37.70%	6.35%	55.95%	
R5 A/H	Yes	8.78%	5.41%	85.81%	0.4834
	No	6.35%	5.56%	88.10%	
R6 A/H	Yes	81.76%	2.03%	16.22%	0.6765
	No	82.54%	6.75%	10.71%	
R7 A/H	Yes	58.78%	6.76%	34.46%	0.3816
	No	62.70%	7.54%	29.76%	
R8 SD/S	Yes	60.81%	4.05%	35.14%	0.0476 *
	No	67.86%	10.71%	21.43%	
R9 SD/S	Yes	34.46%	12.16%	53.38%	0.1910
	No	21.83%	23.02%	55.16%	
R10 SD/S	Yes	82.43%	2.70%	14.86%	0.8548
	No	80.95%	7.14%	11.90%	
R11 A/H	Yes	33.11%	9.46%	57.43%	0.0001 ***
	No	51.59%	12.30%	36.11%	
R12 KS	Yes	45.27%	4.73%	50.00%	0.5863
	No	41.67%	6.35%	51.98%	
R13 KS	Yes	57.43%	2.70%	39.86%	0.2273
	No	50.40%	4.76%	44.84%	
R14 KS	Yes	65.54%	3.38%	31.08%	0.2075
	No	59.13%	3.97%	36.90%	
R15 KS.	Yes	58.11%	3.38%	35.51%	0.0001 ***
	No	29.37%	3.57%	67.06%	
R16 KS	Yes	58.11%	2.03%	39.86%	0.2331
	No	51.19%	3.97%	44.84%	
R17 KS	Yes	64.86%	4.05%	31.08%	0.0859
	No	71.43%	8.33%	20.24%	

* *p* < 0.05 ** *p* < 0.01 *** *p* < 0.001 (Mann–Whitney U test and the Kruskal–Wallis).

## Data Availability

Data available upon request. Please email the lead author.

## Supplementary Materials

The following supporting information can be downloaded at: https://www.mdpi.com/article/10.3390/ijerph22111662/s1, Supplementary Material 1: POSHA-S-CL; Supplementary Material 2: Stuttering Awareness- Busting Myths.

## References

[1] Johnson M., Baxter S., Blank L., Cantrell A., Brumfitt S., Enderby P., Goyder E. (2015). The State of the Art in Non-Pharmacological Interventions for Developmental Stuttering. Part 2: Qualitative Evidence Synthesis of Views and Experiences. Int. J. Lang. Commun. Disord..

[2] Costa J.B., Juste F., Ritto A.P., Sassi F.C., de Andrade C.R.F. (2023). Analysis of Cumulative Risk Predictors for Persistent Stuttering: Family Perception and Amount of Speech Disruptions. Codas.

[3] Sandoval-Fox Y., Román-Zubeldia J., Vázquez-Fernández P. (2024). Methodological Process of Cross-Cultural Adaptation of the Survey The Public Opinion Survey on Human Attributes-Stuttering (POSHA-S) to Spanish-Chilean. Salud Cienc. Tecnol..

[4] Boyle M.P., Daniels D.E., Hughes C.D., Buhr A.P. (2016). Considering disability culture for culturally competent interactions with individuals who stutter. Contemp. Issues Commun. Sci. Disord..

[5] de Britto Pereira M.M., Rossi J.P., Van Borsel J. (2008). Public awareness and knowledge of stuttering in Rio de Janeiro. J. Fluen. Disord..

[6] Boyle M.P. (2017). Personal perceptions and perceived public opinion about stuttering in the United States: Implications for anti-stigma campaigns. Am. J. Speech-Lang. Pathol..

[7] Jokar A.H.R., Roche S., Karimi H. (2023). Stuttering on Instagram: What is the focus of stuttering-related Instagram posts and how do users engage with them?. J. Fluen. Disord..

[8] Sandoval Y., Roman-Zubeldia J., Sacheri S. (2025). Public Opinion Towards Stuttering: The Differentiated Beliefs and Reactions Between Chilean Men and Women. Data Metadata.

[9] Haryani H., Chu S.Y., Yaruss J.S., McConnell G., Ali M.M. (2020). Public Attitudes in Asia Toward Stuttering: A Scoping Review. Open Public Health J..

[10] Rocha M., Yaruss J.S., Rato J.R. (2019). Stuttering Impact: A Shared Perception for Parents and Children?. Folia Phoniatr. Logop..

[11] Amura C.R., Medina R., Bean M., Centi S., Cook P.F., Barton A.J., Jones J. (2024). Socio-Structural Intersect with Post-COVID-19 Telehealth Utilization for Hispanic/Latino Groups in Colorado: A Mixed Methods Study. J. Transcult. Nurs..

[12] Nang C., Hersh D., Milton K., Lau S.R. (2018). The Impact of Stuttering on Development of Self-Identity, Relationships, and Quality of Life in Women Who Stutter. Am. J. Speech-Lang. Pathol..

[13] Piñeiro B., Díaz D.R., Monsalve L.M., Martínez Ú., Meade C.D., Meltzer L.R., Brandon K.O., Unrod M., Brandon T.H., Simmons V.N. (2018). Systematic Transcreation of Self-Help Smoking Cessation Materials for Hispanic/Latino Smokers: Improving Cultural Relevance and Acceptability. J. Health Commun..

[14] Iverach L., Menzies R., Jones M., O'Brian S., Packman A., Onslow M. (2015). Further Development and Validation of the Unhelpful Thoughts and Beliefs About Stuttering (UTBAS) Scales: Relationship to Anxiety and Social Phobia Among Adults Who Stutter. Int. J. Lang. Commun. Disord..

[15] Iverach L., Jones M., Lowe R., O’Brian S., Menzies R.G., Packman A., Onslow M. (2018). Comparison of Adults Who Stutter with and Without Social Anxiety Disorder. J. Fluen. Disord..

[16] Blumgart E., Tran Y., Craig A. (2010). Social anxiety disorder in adults who stutter. Depress. Anxiety.

[17] Tumanova V., Choi D., Conture E.G., Walden T.A. (2018). Expressed Parental Concern Regarding Childhood Stuttering and the Test of Childhood Stuttering. J. Commun. Disord..

[18] Ma Y., Oxley J.D., Yaruss J.S., Tetnowski J.A. (2023). Stuttering experience of people in China: A cross-cultural perspective. J. Fluen. Disord..

[19] Medina A.M., Pareja D., Berlanga E., Crawford C., Tormo A. (2023). Concepts of family support in stuttering: A multicultural perspective. Perspect. ASHA Spec. Interes. Groups.

[20] Millager R.A., Liu T., Pruett D.G., Jones R.M. (2025). A year in stuttering research: A systematic review of global representation and sociodemographic reporting practices in English-language journals in 2020. J. Commun. Disord..

[21] Üstün-Yavuz M.S., Warmington M., Gerlach H., St. Louis K.O. (2021). Cultural difference in attitudes towards stuttering among British, Arab and Chinese students: Considering home and host cultures. Int. J. Lang. Commun. Disord..

[22] Kefalianos E., Onslow M., Ukoumunne O., Block S., Reilly S. (2014). Stuttering, Temperament, and Anxiety: Data from a Community Cohort Ages 2–4 Years. J. Speech Lang. Hear. Res..

[23] Snyder M.C., Arnold H.S. (2022). Emotion-related regulation strategy use in preschool-age children who stutter. J. Commun. Disord..

[24] Johnson G., Onslow M., Horton S., Kefalianos E. (2023). Psychosocial features of stuttering for school-age children: A systematic review. Int. J. Lang. Commun. Disord..

[25] Garabedian A.S. (2024). Temperament and Emotion in Developmental Stuttering: A Meta-Analysis. Master’s Thesis.

[26] Hughes S., Junuzovic-Zunic L., Mostafa E., Weidner M., Özdemir R.S., Daniels D.E., St. Louis K.O. (2024). Mothers’ and fathers’ attitudes toward stuttering in the Middle East compared to Europe and North America. Int. J. Lang. Commun. Disord..

[27] Kulish A.L., Cavanaugh A.M., Stein G.L., Kiang L., Gonzalez L.M., Supple A., Mejia Y. (2019). Ethnic–Racial Socialization in Latino Families: The Influence of Mothers’ Socialization Practices on Adolescent Private Regard, Familism, and Perceived Ethnic–Racial Discrimination. Cult. Divers. Ethn. Minor. Psychol..

[28] Lefort M.K., Erickson S., Block S., Carey B., St. Louis K.O. (2021). Australian attitudes towards stuttering: A cross-sectional study. J. Fluen. Disord..

[29] Dean L., Medina A.M. (2021). Stigma and the Hispanic stuttering experience: A qualitative study. J. Commun. Disord..

[30] Medina A.M., Mead J.S., Moore S. (2024). Perceptions of and beliefs about stuttering in the Hispanic/Latino community. J. Commun. Disord..

[31] Isaacs D.H. (2021). Satan is holding your tongue back: Stuttering as moral failure. Afr. J. Disabil..

[32] Isaacs D., Swartz L. (2022). Examining the understandings of young adult South African men who stutter: The question of disability. Int. J. Lang. Commun. Disord..

[33] Bellegarde M., Mayo R., St. Louis K.O., Mayo C.M. (2016). Public opinions of stuttering in Haiti. ECHO J. Natl. Black Assoc. Speech-Lang. Hear..

[34] Sandoval Y., García V., Sanhueza M. (2022). Perception of People with Stuttering Regarding their Experiences with Interventions Based on the CALMS Multidimensional Model. Rev. Chil. Fonoaudiol..

[35] Pino K.M. (2024). Telepráctica para el abordaje de la tartamudez. Tartamudez: Abordaje Fonoaudiológico a Partir del Modelo Calms.

[36] Boyle M.P. (2015). Relationships between psychosocial factors and quality of life for adults who stutter. Am. J. Speech-Lang. Pathol..

[37] Ríos A., López-Navas A., Ayala-García M.A., Sebastián M.J., Abdo-Cuza A., Martínez-Alarcón L., Ramírez E.J., Muñoz G., Palacios G., Suárez-López J. (2014). The Attitude Toward Living Kidney Donation Among Personnel from Units Related to Donation and Transplantation in Spain, Mexico and Cuba. Ren. Fail..

[38] Feldman O., Goldstien E., Rolnik B., Ganz A.B., Lev-Ari S. (2021). Inquiry based stress reduction (IBSR) improves overall stuttering experience among adults who stutter: A randomized controlled trial. J. Clin. Med..

[39] Iverach L., Jones M., McLellan L.F., Lyneham H.J., Menzies R.G., Onslow M., Rapee R.M. (2016). Prevalence of Anxiety Disorders Among Children Who Stutter. J. Fluen. Disord..

[40] Beilby J.M., Byrnes M.L., Yaruss J.S. (2012). Acceptance and Commitment Therapy for Adults Who Stutter: Psychosocial Adjustment and Speech Fluency. J. Fluen. Disord..

[41] St. Louis K.O., Reichel I.K., Yaruss J.S., Lubker B.B. (2009). Construct and Concurrent Validity of a Prototype Questionnaire to Survey Public Attitudes Toward Stuttering. J. Fluen. Disord..

[42] Nonis D., Unicomb R., Hewat S. (2022). Parental perceptions of stuttering in children: A systematic review of the literature. Speech Lang. Hear..

[43] St. Louis K.O., Sønsterud H., Junuzović-Žunić L., Tomaiuoli D., Del Gado F., Caparelli E., Theiling M., Flobakk C., Helmen L.N., Heitmann R.R. (2016). Public attitudes toward stuttering in Europe: Within-country and between-country comparisons. J. Commun. Disord..

[44] St. Louis K.O., Öge-Daşdöğen Ö., Johnson L.E., Litzinger J.G., Myers L., Donai J. (2022). Relationships Between Public Attitudes Toward Stuttering and Autonomic and Subjective Indices of Anxiety. Forum Lingwist..

[45] Davis R.E., Johnson T.P., Lee S., Werner C. (2018). Why Do Latino Survey Respondents Acquiesce? Respondent and Interviewer Characteristics as Determinants of Cultural Patterns of Acquiescence Among Latino Survey Respondents. Cross-Cult. Res..

[46] Lakens D. (2022). Sample size justification. Collabra Psychol..

[47] Soroya S.H., Rehman A.U., Faiola A. (2024). Exploring the impact of Internet and media sources exposure on self-care behavior: Mediating the role of health anxiety, literacy and information-seeking behavior. Kybernetes.

[48] Rattay P., Von Der Lippe E., Mauz E., Richter F., Hölling H., Lange C., Lampert T. (2018). Health and Health Risk Behaviour of Adolescents—Differences According to Family Structure. Results of the German KiGGS Cohort Study. PLoS ONE.

[49] Umberson D., Montez J.K. (2010). Social relationships and health: A flashpoint for health policy. J. Health Soc. Behav..

[50] Putland C., Baum F., Ziersch A., Arthurson K., Pomagalska D. (2013). Enabling pathways to health equity: Developing a framework for implementing social capital in practice. BMC Public Health.

[51] Wilson D.K., Sweeney A.M., Kitzman-Ulrich H., Gause H., George S.M.S. (2017). Promoting social nurturance and positive social environments to reduce obesity in high-risk youth. Clin. Child. Fam. Psychol. Rev..

[52] Mancini J.A., O'Neal C.W., Martin J.A., Bowen G.L. (2018). Community social organization and military families: Theoretical perspectives on transitions, contexts, and resilience. J. Fam. Theory Rev..

[53] Arnold H.S., Li J. (2016). Associations between beliefs about and reactions toward people who stutter. J. Fluen. Disord..

[54] Hughes C.D., Gabel R.M., Goberman A. (2017). Examining the relationship between perceptions of a known versus average person who stutters. Can. J. Speech-Lang. Pathol. Audiol..

[55] Gabel R.M. (2011). Family experiences of people who stutter. Can. J. Speech-Lang. Pathol. Audiol..

[56] Afzal A., Noor H.S., Rubab M., Arshad S., Saleem A. (2023). Comparative Study in Adults Who Stutter with and Without Social Anxiety. J. Health Res..

[57] Zeigler-Hill V., Besser A., Besser Y. (2021). The Negative Consequences of Stuttering for Perceptions of Leadership Ability. J. Individ. Differ..

[58] Connery A., Galvin R., McCurtin A. (2021). Effectiveness of Nonpharmacological Stuttering Interventions on Communication and Psychosocial Functioning in Adults: A Systematic Review and Meta-Analysis of Randomized Controlled Trials. J. Evid.-Based Med..

[59] De Sonneville-Koedoot C., Stolk E., Rietveld T., Franken M.-C. (2015). Direct Versus Indirect Treatment for Preschool Children Who Stutter: The RESTART Randomized Trial. PLoS ONE.

[60] Uysal A.A., Tura G. (2018). Candidate Teachers’ Attitude and Knowledge Toward Speech and Language Disorders in Children. Kocaeli Üniversitesi Eğitim Dergisi.

[61] Below J., Polikowsky H., Scartozzi A., Shaw D., Pruett D., Chen H.-H., Petty L., Petty A., Lowther E., Yu Y. (2023). Discovery of 36 Loci Significantly Associated with Stuttering. Res. Square.

[62] Obiweluozo P.E., Ede M.O., Onwurah C.N., Uzodinma U.E., Dike I.C., Ejiofor J.N. (2021). Impact of Cognitive Behavioral Play Therapy on Social Anxiety Among School Children with Stuttering Deficit: A Cluster Randomized Trial with Three Months Follow-Up. Medicine.

[63] Haitani T. (2022). A Multilevel Meta-Analysis on Effects of Psychological Interventions for Adults Who Stutter. OSF Prepr..

