# Supplementary material for: Influence of Marital and Parental Status on Public Reactions to Stuttering in Chile: A Socio-Demographic Study
Source: Int J Environ Res Public Health. 2025 Nov 2;22(11):1662. doi: 10.3390/ijerph22111662 (PMC12652990; doi:10.3390/ijerph22111662)
Supplement: Supplementary file 1 [file ijerph-22-01662-s001.zip › Supplementary Material 1. POSHA-S-CL.pdf]

Instrucciones
Estimado Participante,

Se agradece su participación en este proyecto de investigación diseñado para explorar la opinión pública sobre una serie de atributos y características humanas en varios lugares del mundo. La siguiente encuesta le pide opiniones honestas sobre cinco distintos atributos humanos y cierta información sobre usted para ayudar a interpretar mejor los resultados. La encuesta también solicita opiniones más detalladas sobre uno de los atributos humanos.

Por favor, no escriba su nombre, dirección o número de teléfono en ninguna parte de la encuesta o en el sobre utilizado para enviarlo. Es importante que su nombre no esté incluido para que se pueda mantener una confidencialidad completa

Contestar esta encuesta completamente ayudará a proporcionar una imagen más clara de la opinión pública. Sin embargo, a medida que complete la encuesta, puede omitir cualquier elemento o dejar de responder por cualquier motivo, sin perjuicio ni penalización.

La encuesta solicita algunas respuestas cortas escritas y seleccionar algunas casillas de verificación [θ] que se aplican a usted, pero en su mayoría implica hacer juicios haciendo un **círculo alrededor de su respuesta**. Algunos de estos juicios son números en escalas numéricas, mientras que otros son opciones "Sí", "No" o "No estoy seguro". ¡No hay respuestas correctas o incorrectas! *Le pedimos que trabaje rápidamente y marque su primera impresión.* No regrese y cambie ninguna de sus respuestas a menos que más tarde descubra que no entendió un elemento o que respondió en la línea incorrecta.

Cuando dé su opinión, asegúrese de **hacer un pequeño círculo** alrededor del número, pregunta o palabra que **mejor represente su opinión**. En las escalas numéricas, puede marcar con un círculo cualquier número, pero siéntase libre de marcar los extremos negativos o positivos de la escala, así como el centro exacto si uno de esos muestra su opinión. Cuando marque una casilla, coloque una pequeña ✓ en la casilla.

A continuación, hay cuatro ejemplos de respuestas. El primero muestra una opinión bastante positiva sobre alguien que es de *estatura alta*, el segundo una opinión muy negativa sobre alguien que es de *estatura baja*, una opinión neutra sobre alguien que *usa anteojos*, y no tiene opinión o no sabe sobre alguien que *use de un audífono para escuchar*.

**Mi impresión general de una persona que…**	**Muy negativo**	**Algo negativo**	**Neutro**	**Algo positivo**	**Muy positivo**	**Inseguro(a)**
es de estatura alta	**1**	**2**	**3**	**(4)**	**5**	**?**
es de estatura baja	**(1)**	**2**	**3**	**4**	**5**	**?**
usa lentes	**1**	**2**	**(3)**	**4**	**5**	**?**
usa un audífono para escuchar	**1**	**2**	**3**	**4**	**5**	**(?)**

Muchas gracias por su cooperación.

Encuesta de opinión pública de atributos humanos-tartamudez. **Por favor cuéntenos sobre usted en esta sección.**

**Fecha:**	**Día**	**Mes**	**Año**
Fecha de hoy:	Ej.:23<br>________________	Ej.: Enero<br>_____	Ej.: 2017<br>___________________
La fecha en que nací			

**Residencia y ciudadanía**	**País**	**Región**	**Cuidad/Comuna**
Ahora vivo en:	________________________	________________________	________________________
Nací en:			

Seleccione [✓] todas las opciones que aplican

**Soy**: θHombreθMujer	**Estoy/He estado casado/a:**θSiθ No	**Soy/He sido padre/madre:**θ Siθ No

**He completado los siguientes niveles escolares:**	
θBásica (8 años de educación)	θTécnico (3 años de educación)
θ Media (4 años de educación)	θUniversitario (5 años de educación)
θ Otro	θ Magister o post grado (≥18 años de educación)

**Situación laboral:**	
θEstudiante escuela /universidad	θDesempleado o sin trabajo
θTrabajando	θJubilado/a

El trabajo que estoy mejor capacitado para hacer, o el trabajo en el que trabajé más tiempo es (era)

____

____

____

Mi lengua materna es

También puedo **entender y hablar** fácilmente los siguientes idiomas:

1.	2.	3.
______________________	______________________	______________________
______________________	______________________	______________________

Encierre en un círculo el número o no estoy seguro/a (?) al lado de cada característica o marque [✓] las casillas que correspondan.

**Los ingresos de mi familia son […] en comparación a los ingresos anuales de…**	**Más bajos**		**Promedio**		**Más altos**	**No estoy seguro/a**
mis familiares y mis amigos	**1**	**2**	**3**	**4**	**5**	**?**
toda la gente de mi país	**1**	**2**	**3**	**4**	**5**	**?**

Mi religión es:	

**Calificaría los siguientes aspectos de mi vida ahora como ...**	**Muy pobre**	**Pobre**	**Promedio**	**Bueno**	**Excelente**	**No estoy seguro/a**
mi salud física	**1**	**2**	**3**	**4**	**5**	**?**
mi salud mental	**1**	**2**	**3**	**4**	**5**	**?**
mi habilidad para aprender cosas nuevas	**1**	**2**	**3**	**4**	**5**	**?**
mi habilidad para hablar	**1**	**2**	**3**	**4**	**5**	**?**

**Que tan importante son para mí los siguientes aspectos…**	**Nunca importante**	**Normalmente no importante**	**Igual de importante o no importante**	**Normalmente importante**	**Siempre importante**	**No estoy seguro/a**
estar a salvo y seguro	**1**	**2**	**3**	**4**	**5**	**?**
ser libre de hacer lo que quiera	**1**	**2**	**3**	**4**	**5**	**?**
pasar tiempo tranquilo solo/a	**1**	**2**	**3**	**4**	**5**	**?**
asistir a fiestas o eventos sociales	**1**	**2**	**3**	**4**	**5**	**?**
imaginar cosas nuevas	**1**	**2**	**3**	**4**	**5**	**?**
ayudar a los menos afortunados	**1**	**2**	**3**	**4**	**5**	**?**
tener experiencias emocionantes pero potencialmente "peligrosas"	**1**	**2**	**3**	**4**	**5**	**?**
practicar mi religión	**1**	**2**	**3**	**4**	**5**	**?**
ganar dinero	**1**	**2**	**3**	**4**	**5**	**?**
hacer mi trabajo o mis deberes	**1**	**2**	**3**	**4**	**5**	**?**
terminar las cosas	**1**	**2**	**3**	**4**	**5**	**?**
averiguar cómo resolver problemas importantes	**1**	**2**	**3**	**4**	**5**	**?**

Ahora, por favor denos su opinión sobre las <u>personas con todas las características enumeradas</u>.

**Mi impresión general de una persona que…**	**Muy negativa**	**Algo negativa**	**Neutra**	**Algo positiva**	**Muy positiva**	**No estoy seguro/a**
es obeso/a (con mucho sobrepeso)	**-2**	**-1**	**0**	**+1**	**+2**	**?**
es zurdo/a	**-2**	**-1**	**0**	**+1**	**+2**	**?**
tiene tartamudez	**-2**	**-1**	**0**	**+1**	**+2**	**?**
tiene una enfermedad mental	**-2**	**-1**	**0**	**+1**	**+2**	**?**
es inteligente	**-2**	**-1**	**0**	**+1**	**+2**	**?**

**Me gustaría ser una persona que ...**	**Muy en desacuerdo**	**Algo desacuerdo**	**Neutro**	**Algo de acuerdo**	**Muy de acuerdo**	**No estoy seguro/a**
es obeso/a (con mucho sobrepeso)	**-2**	**-1**	**0**	**+1**	**+2**	**?**
es zurdo/a	**-2**	**-1**	**0**	**+1**	**+2**	**?**
tiene tartamudez	**-2**	**-1**	**0**	**+1**	**+2**	**?**
tiene una enfermedad mental	**-2**	**-1**	**0**	**+1**	**+2**	**?**
es inteligente	**-2**	**-1**	**0**	**+1**	**+2**	**?**

**Cuánto sé de una persona que es…**	**Nada**	**Poco**	**Algo**	**Mucho**	**Una gran cantidad**	**No estoy seguro/a**
es obeso/a (con mucho sobrepeso)	**1**	**2**	**3**	**4**	**5**	**?**
es zurdo/a	**1**	**2**	**3**	**4**	**5**	**?**
tiene tartamudez	**1**	**2**	**3**	**4**	**5**	**?**
tiene una enfermedad mental	**1**	**2**	**3**	**4**	**5**	**?**
es inteligente	**1**	**2**	**3**	**4**	**5**	**?**

Ahora, por favor denos opiniones más detalladas sobre el <u>trastorno de la tartamudez</u>.

**<u>Las personas que tartamudean ...</u>**			**No estoy seguro/a**
deberían tratar de ocultar su tartamudeo	**Si**	**No**	**?**
deberían tener trabajos donde tengan que entender y decidir correctamente las cosas importantes	**Si**	**No**	**?**
son nerviosos o fáciles de emocionar	**Si**	**No**	**?**
son tímidos o temerosos	**Si**	**No**	**?**
tienen la culpa de ser tartamudos	**Si**	**No**	**?**
pueden hacer amigos	**Si**	**No**	**?**
pueden llevar vidas normales	**Si**	**No**	**?**
pueden hacer cualquier trabajo que quieran	**Si**	**No**	**?**

<u>**Estaría preocupado/a**</u> **si las siguientes personas tartamudearan…**			**No estoy seguro/a**
mi doctor	**Si**	**No**	¿
mi vecino/a	**Si**	**No**	¿
mi hermano/a	**Si**	**No**	¿
Yo	**Si**	**No**	¿

**Si estuviera hablando con una persona que tartamudea, yo …**			**No estoy seguro/a**
Trataría de actuar como si la persona estuviera hablando normalmente	**Si**	**No**	¿
haría una broma sobre la tartamudez	**Si**	**No**	¿
completaría las palabras de la persona	**Si**	**No**	¿
me sentiría impaciente (no quisiera esperar mientras la persona tartamudea)	**Si**	**No**	¿
me sentiría relajado/a o cómodo/a	**Si**	**No**	¿
sentiría lástima por la persona	**Si**	**No**	¿
le diría a la persona que "fuera más lento" o "se relajara"	**Si**	**No**	¿

**Creo que la tartamudez es causada por …**			**No estoy seguro/a**
herencia genética	**Si**	**No**	¿
fantasmas, demonios o espíritus	**Si**	**No**	¿
un evento traumático	**Si**	**No**	¿
un acto de Dios	**Si**	**No**	¿
Aprendizaje o habito	**Si**	**No**	¿
un virus o una enfermedad	**Si**	**No**	¿

**Creo que la tartamudez debería ser ayudada por ...**			**No estoy seguro/a**
otras personas que tartamudean	**Si**	**No**	**?**
Fonoaudiólogos	**Si**	**No**	**?**
gente como yo	**Si**	**No**	**?**
un doctor	**Si**	**No**	**?**

**Mi conocimiento de la tartamudez viene de ...**			**No estoy seguro/a**
mi experiencia personal (yo, mi familia, amigos)	**Si**	**No**	**?**
la televisión, radio, o películas	**Si**	**No**	**?**
revistas, periódicos, o libros	**Si**	**No**	**?**
Internet	**Si**	**No**	**?**
mi educación (escolar, académica)	**Si**	**No**	**?**
doctores, enfermeros/as, y otros especialistas	**Si**	**No**	**?**

¡Ha terminado! Muchas gracias.
¿Cuánto tiempo se demoró completar la encuesta? _____Minutos

Table: Reactions (R) section POSHA-S-CL. Includes social distance/sympathy (SD/S); accommodation/help (A/H); knowledge source (KS)

Code	Question
R1 SD/S	Estaría preocupado si la siguiente persona presentara tartamudez ... "mi doctor". I would be concerned if the following person had a stutter … "my doctor."
R2 SD/S	Estaría preocupado si la siguiente persona presentara tartamudez ... "mi vecino". I would be concerned if the following person had a stutter … "my neighbor."
R3 SD/S	Estaría preocupado si la siguiente persona presentara tartamudez ... "mi hermano/a". I would be concerned if the following person had a stutter … "my brother/sister."
R4 SD/S	Estaría preocupado si la siguiente persona presentara tartamudez ... "yo". I would be concerned if the following person had a stutter … "me."
R8 SD/S	Si estuviera hablando con una persona que presenta tartamudez, yo me sentiría impaciente. If I were talking with someone who stutters, I would feel impatient.
R9 SD/S	Si estuviera hablando con una persona que presenta tartamudez, yo me sentiría relajado/a o cómodo/a. If I were talking with someone who stutters, I would feel relaxed or comfortable.
R10 SD/S	Si estuviera hablando con una persona que presenta tartamudez, yo sentiría lástima por esa persona. If I were talking with someone who stutters, I would feel pity for that person.
R5 A/H	Si estuviera hablando con una persona que presenta tartamudez, yo trataría de actuar como si la persona estuviera hablando normalmente. If I were talking with someone who stutters, I would try to act as if that person were speaking normally.
R6 A/H	Si estuviera hablando con una persona que presenta tartamudez, yo haría una broma sobre la tartamudez. If I were talking with someone who stutters, I would make a joke about stuttering.
R7 A/H	Si estuviera hablando con una persona que presenta tartamudez, yo completaría las palabras de la persona. If I were talking with someone who stutters, I would complete the person's words.
R11 A/H	Si estuviera hablando con una persona que presenta tartamudez, yo le diría a la persona que "fuera más lento" o "se relajara". If I were talking with someone who stutters, I would tell the person to "speak more slowly" or "relax."
R12 KS	Mi conocimiento de la tartamudez viene de... mi experiencia personal (yo, mi familia, amigos). My knowledge of stuttering comes from… personal experience (me, my family, friends).
R13 KS	Mi conocimiento de la tartamudez viene de la televisión, radio o películas. My knowledge of stuttering comes from television, radio, or movies.
R14 KS	Mi conocimiento de la tartamudez viene de revistas, periódicos o libros. My knowledge of stuttering comes from magazines, newspapers, or books.
R15 KS	Mi conocimiento de la tartamudez viene de internet. My knowledge of stuttering comes from the internet..
R16 KS	Mi conocimiento de la tartamudez viene de mi educación (escolar, académica). My knowledge of stuttering comes from my education (school, academia).
R17 KS	Mi conocimiento de la tartamudez viene de doctores, especialistas enfermeros/as y otros. My knowledge of stuttering comes from doctors, specialists, nurses, and others.
