# Supplementary material for: Influence of Marital and Parental Status on Public Reactions to Stuttering in Chile: A Socio-Demographic Study
Source: Int J Environ Res Public Health. 2025 Nov 2;22(11):1662. doi: 10.3390/ijerph22111662 (PMC12652990; doi:10.3390/ijerph22111662)
Supplement: Supplementary file 1 [file ijerph-22-01662-s001.zip › Supplementary Material 2. Stuttering Awareness- Busting Myths.pdf]

Concientización de la tartamudez: Derribando Mitos

La tartamudez ha estado rodeada de mitos y creencias que debemos erradicar. A través de la difusión de información veraz, esperamos derribar el estigma social que acompaña esta forma distinta de hablar.

1 LAS PERSONAS QUE TARTAMUDEAN SON MENOS INTELIGENTES

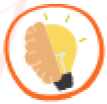

Algunas personas confunden la falta de fluidez al expresar las ideas con una menor inteligencia, sin embargo, no existe una relación directa entre ambas habilidades. La persona que tartamudea tiene las mismas habilidades que quienes hablan "fluido".

2 LA TARTAMUDEZ ES CAUSADA POR NERVIOS

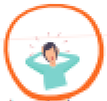

Recientes estudios dan cuenta del origen neurobiológico de la tartamudez, descartando que su causa sea por nerviosismo. Sin embargo, quienes tartamudean pueden incrementar sus síntomas ante situaciones estresantes asociadas al habla como exposiciones orales, entrevistas, llamadas telefónicas, leer en voz alta, entre otras.

3 LA TARTAMUDEZ PUEDE SER APRENDIDA

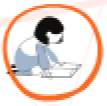

La tartamudez es involuntaria, por lo mismo, no puede ser aprendida. Si bien la tartamudez aparece entre familiares, es debido a un factor genético y no a la imitación, ya que es muy incómoda para quien la vive, resultando imposible mantenerla en el tiempo.

4 ES NORMAL QUE LOS NIÑOS TARTAMUDEEN CUANDO SON PEQUEÑOS

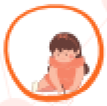

Es frecuente que existan algunas disfluencias cuando los niños están aprendiendo a utilizar el lenguaje, sin embargo, la tartamudez puede aparecer en la misma etapa. Es importante que un especialista evalúe en el habla del niño las características e indicadores que permitirán determinar el tipo de intervención más apropiada.

5 CUANDO UNA PERSONA SE TRABA HAY QUE AYUDARLO DICIÉNDOLE "PIENSA ANTES DE HABLAR", "RESPIRA", "CÁLMATE" O "RELÁJATE"

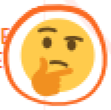

Haciendo este tipo de comentarios, no se está poniendo atención a lo que la persona dice, sino que a su tartamudez. Como la tartamudez no es voluntaria ni se produce por nerviosismo, estos comentarios, podrían exacerbar las trabas durante la conversación.

6 LAS PERSONAS TARTAMUDEAN PARA LLAMAR LA ATENCIÓN

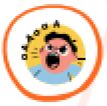

La tartamudez es involuntaria e incómoda para quien la vive, por lo tanto no es agradable sostenerla en el tiempo para obtener la atención de otros.

7 PARA DEJAR DE TARTAMUDEAR, BASTA CON TENER FUERZA DE VOLUNTAD

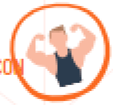

Como la tartamudez es involuntaria; quien tartamudea tiene dificultad para lograr un control absoluto de los movimientos de su habla, por lo tanto no basta con esforzarse para que la tartamudez deje de aparecer. Ahora bien, la fuerza de voluntad, resultará un factor indispensable al momento de iniciar el tratamiento con un especialista.

8 LA TARTAMUDEZ APARECE POR CULPADE LOS PADRES

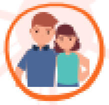

Como ya hemos mencionado, esta condición tiene base neurobiológica, no es causada por los padres, sin embargo, los factores ambientales influyen en la experiencia del niño con la tartamudez y podrían contribuir a dificultades en su comunicación. Es por ello que cobra real importancia el que la familia se involucre en la terapia para realizar los cambios que ayuden al niño. (Early childhood stuttering therapy: A practical guide (Yaruss & Reeves, 2017)

9 EN LOS ADULTOS QUE TARTAMUDEAN, NO HAY NADA QUE HACER

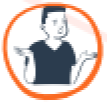

La tartamudez no tiene cura, pero mediante un abordaje integral de la tartamudez, se puede lograr una mayor fluidez y control de los momentos de tartamudez, así como una experiencia comunicativa más cómoda.

10 PARA DIAGNOSTICAR LA TARTAMUDEZ, SE DEBE ESPERAR HASTA LOS 6 AÑOS

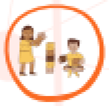

La tartamudez puede diagnosticarse tempranamente. Cuanto más cerca de su aparición se intervenga, mejor es el pronóstico.

11 LA TARTAMUDEZ ES PSICOLÓGICA

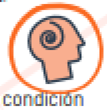

Cuando se ve afectada la expresión oral por una condición como la tartamudez, es muy probable que las experiencias comunicativas puedan verse acompañadas de pensamientos y sentimientos negativos. Por lo tanto el compromiso psicológico que puede darse en la tartamudez no es la causa, sino una consecuencia de las experiencias vividas. Es así como en algunos casos la asesoría psicológica resultará de gran apoyo a la terapia fonoaudiológica en el abordaje de la tartamudez.

12 NO SE DEBE HABLAR DE TARTAMUDEZ FRENTE A LA PERSONA

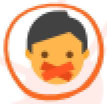

Es importante hablar de la tartamudez con naturalidad. No es cierto que la conducta se fija en los niños cuando hablamos sobre lo que les pasa, al contrario, para ellos es bueno saber que los aceptamos incondicionalmente y valoramos su comunicación.
